# The Implications of Fragmented Genomic DNA Size Range on the Hybridization Efficiency in NanoGene Assay

**DOI:** 10.3390/s18082646

**Published:** 2018-08-13

**Authors:** Xiaofang Wang, Beelee Chua, Ahjeong Son

**Affiliations:** 1 Department of Civil Engineering, Auburn University, Auburn, AL 36849, USA; emily.xfwang@gmail.com; 2 School of Electrical Engineering, Korea University, Seoul 02841, Korea; bchua@korea.ac.kr; 3 Department of Environmental Science and Engineering, Ewha Womans University, Seoul 03760, Korea

**Keywords:** DNA fragmentation, optimum size, quantification capability, hybridization efficiency, magnetic beads, quantum dots

## Abstract

DNA hybridization-based assays are well known for their ability to detect and quantify specific bacteria. Assays that employ DNA hybridization include a NanoGene assay, fluorescence in situ hybridization, and microarrays. Involved in DNA hybridization, fragmentation of genomic DNA (gDNA) is necessary to increase the accessibility of the probe DNA to the target gDNA. However, there has been no thorough and systematic characterization of different fragmented gDNA sizes and their effects on hybridization efficiency. An optimum fragmented size range of gDNA for the NanoGene assay is hypothesized in this study. Bacterial gDNA is fragmented via sonication into different size ranges prior to the NanoGene assay. The optimum size range of gDNA is determined via the comparison of respective hybridization efficiencies (in the form of quantification capabilities). Different incubation durations are also investigated. Finally, the quantification capability of the fragmented (at optimum size range) and unfragmented gDNA is compared.

## 1. Introduction

DNA hybridization is a well-known and critical process in assays that targets specific genomic DNA (gDNA). Essentially, it is the formation of a hydrogen bond between complementary sequences of probe single-stranded DNA (ssDNA) and the target ssDNA. It is employed widely in numerous bioassays, such as membrane hybridization [1,2,3], fluorescence in situ hybridization (FISH) [4,5,6,7], microarray hybridization [8,9,10,11], and electrochemical DNA assays [12,13,14,15]. It is also used in magnetic beads and the quantum dots-based NanoGene assay [16,17].

The target gDNA can be from a variety of sources, such as microorganisms (like pathogenic bacteria) to human body analytes (such as blood). Since the gDNA is double-stranded and has a large supramolecular structure, it needs to be fragmented (as a pretreatment) prior to hybridization with the probe ssDNA. The fragmentation of long double-stranded gDNA into shorter gDNA will increase its accessibility to the probe ssDNA and, hence, allow for the achievement of higher hybridization efficiency.

Characterization of pretreatment conditions to determine optimum environments for a particular assay is a mundane but, unfortunately, a very important exercise. It enables the particular assay to subsequently perform at its optimum conditions in its respective applications, such as identification of pathogenic bacteria. This study is no exception. Specifically, the authors are interested in the existence of an optimum fragmented gDNA size range for the NanoGene assay. The NanoGene assay has previously demonstrated its ability to detect and quantify specific bacteria (both airborne and waterborne) based on their gDNA. Furthermore, it could be implemented via portable apparatus, thus enabling the possibility for mobile monitoring as well as point-of-investigation diagnostics [17,18,19,20].

The NanoGene assay uses particle complexes consisting of magnetic beads, a set of quantum dots, and probe and signaling DNAs. Both probe and signaling DNA complexes can be tethered together via DNA hybridization with the target gDNA. Magnetic beads are used as carriers that are suitable for magnetic manipulation. The normalized fluorescence intensity from a set of quantum dots will be indicative of the amount of target gDNA. The normalized fluorescence also depends strongly on the hybridization efficiency (% of target gDNA hybridized with probe and signaling complexes). Therefore, in the absence of photobleaching and quenching while all else being equal, the hybridization efficiency is also representative of the NanoGene assay’s quantification capability and vice versa.

During this study, the gDNA of the target bacterium (*Pseudomonas putida)* is first extracted, followed by sonication for DNA fragmentation. The authors have shown previously that sonication is the most efficient method for gDNA fragmentation with sizes ranging from ~200–1000 base pairs or bp [21]. Following sonication, the fragmented gDNA of various sizes are analyzed using the NanoGene assay. The respective NanoGene assay normalized fluorescence intensity (hence, quantification capabilities) is compared, and the optimum size range is identified. Different hybridization durations, using the optimum fragmented gDNA size range, are also characterized. Finally, the fragmented (at optimum size range) and unfragmented gDNA are also analyzed again in the NanoGene assay, and the difference in their quantification capabilities is compared.

## 2. Materials and Methods

### 2.1. Bacterial Cell Culture and gDNA Extraction

*Pseudomonas putida* strain DSM 8368 (DSMZ; Braunschweig, Germany) was selected as a model bacterium for this study. The dry cells were revived in 1 mL of the tryptic soy broth (Difco Laboratories, Detroit, MI, USA) in a Gallenkamp orbital shaker at 300 rpm at ambient temperature for 5 days. Afterward, the cells were streaked on a tryptic soy agar (Difco) plate and a single colony was chosen. The single colony inoculated in the tryptic soy broth (Difco) was incubated in a Gallenkamp orbital shaker at 300 rpm at ambient temperature for 5 days to achieve a stationary phase of growth (OD ≅ 1.2). The growth of the bacteria was monitored by measuring the optical density at 600 nm using a SpecraMax^®^ M2 Multi-Mode Microplate Reader (Molecular Devices, Sunnyvale, CA, USA). Later, the bacteria were collected from 1 L medium via centrifugation at 5000× *g* for 30 min (AccuSpin^TM^ 400, Fisher Scientific, Waltham, MA, USA). The pellet was subjected to gDNA extraction using a FastDNA^®^ SPIN Kit for Soil (MP Biomedicals, Solon, OH, USA), followed by additional purification using Zymo Genomic DNA Clean and Concentrator^TM^ (Zymo Research, Irvine, CA, USA). The quantity and the purity of the extracted gDNA were determined via the NanoDrop^TM^ 1000 spectrophotometer (Fisher Scientific, Pittsburg, PA, USA).

### 2.2. Preparation and Sizing of Fragmented gDNA Samples

Sonication was performed via XL-2000 Ultrasonic Dismembrator with a P-3 microprobe (Qsonica, Newtown, CT, USA) for 10 s. The output power of the sonicator was set to 10 W. The samples were placed on ice to minimize heat generation during sonication. The microprobe was washed three times with 70% ethanol and a Kimwipes^®^ tissue. Subsequently, the gDNA fragments were subjected to agarose gel electrophoresis for sizing. The fragmented gDNA was loaded on a 2% agarose gel, and 110 V was applied for 1 h (Biorad, Hercules, CA, USA), followed by visualization with a UV Transilluminator (Fisher Scientific, Pittsburg, PA, USA). The size of the fragmented gDNA was measured against a 1 kb ladder (Biorad, Hercules, CA, USA). Subsequently, the electrophoresis gel was excised manually for each size range: 200–500 bp, 500–1000 bp, 1000–1500 bp, 1500–2000 bp, 2000–2500 bp, and 2500–3000 bp. The respective fragmented gDNA for each size range was obtained from the excised gel using the Zymoclean^TM^ Gel Recovery kit (Zymo Research). To validate, the fragmented gDNA was visualized with 2% agarose gel electrophoresis.

### 2.3. Quantification Capability of NanoGene Assay Using Fragmented gDNA of Different Size Ranges

**Figure 1:**
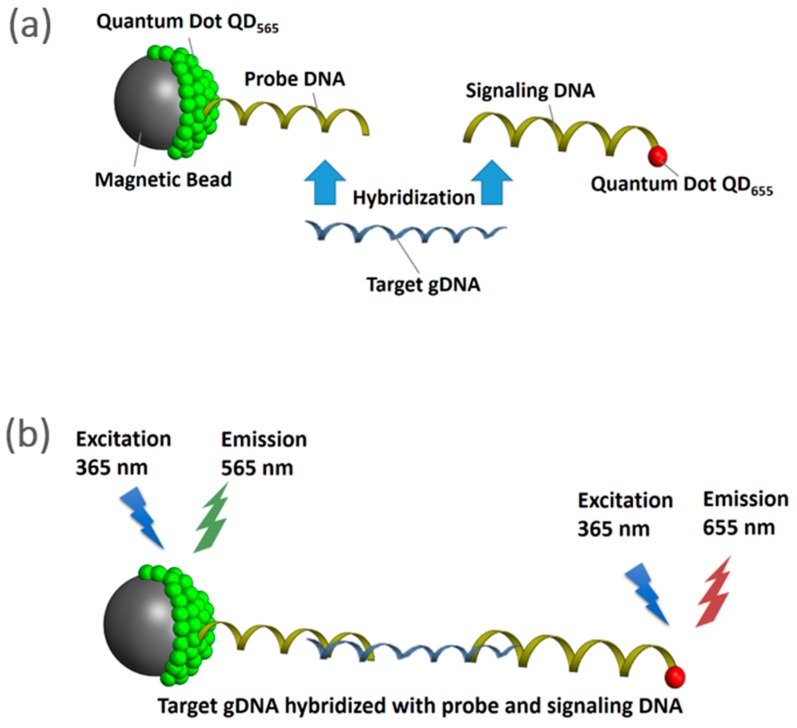
Schematics of the NanoGene assay (**a**) comprised of magnetic beads, quantum dots, and DNA hybridization (**b**) fluorescence measurement.

Briefly, as shown in Figure 1, the NanoGene assay uses dual complexes consisting of (i) probe complex: magnetic beads, quantum dots (emission 565 nm) and probe DNA, and (ii) signaling complex: signaling DNA and quantum dots (emission 655 nm). Both probe and signaling complexes can be tethered together via hybridization with the target gDNA. Fluorescence measurement is performed while exciting the tethered complexes with a 360 nm light source. Thus, the normalized fluorescence intensity (ratio of emission at 655 nm–565 nm) of the NanoGene assay will be proportional to the amount of target gDNA.

The PAH-RHD_α_ (alpha subunit of the PAH-ring hydroxylating dioxygenases) gene was selected as a target gene for the quantification of *P. putida*. The PAH-RHD_α_ gene is involved in the initial step of the PAHs metabolism by the *P. putida* [22]. The quantification of *P. putida* by the NanoGene assay was performed based on the protocol previously developed by Kim and Son [16].

(i) Preparation of probe complex: 8 µL of 2 µmol/L Qdot^®^ 565 ITK™ carboxyl quantum dot nanoparticles (QD_565_, Invitrogen, Carlsbad, CA, USA) were immobilized on the surface of 100 µL of 10^7^ beads/mL aminated Dynabeads^®^ M-270 magnetic beads (MB, Invitrogen, Carlsbad, CA, USA). Dynabeads^®^ M-270 MB with 2.8 µm in diameter are superparamagnetic, and have a monolayer of amine functional group on their surface. This was followed by conjugating 5 µL of 100 nmol/L aminated probe DNA (5′-NH_2_-C_6_-AAG CTG TTG TTC GGG AAG ARW GTG C-3′, IDT, Coralville, IA, USA) on the surface of QD_565_ by forming an amide bond via the reaction of ethylcarbodiimide hydrochloride (EDC, Sigma-Aldrich, St. Louis, MO, USA) and *N*-hydroxysuccinimide (NHS, Sigma-Aldrich, St. Louis, MO, USA).
(ii) Preparation of signaling complex: Aliquots (1.6 µL) of 100 nmol/L aminated signaling probe DNA (5′-CAA CCM ACG TGG TAT GCA TCT CAT C-NH_2_-3′) were attached covalently to the surface of 8 µL of 2 µmol/L carboxyl quantum dots (Qdot^®^ 655 ITK™, QD_655_, Invitrogen, Carlsbad, CA, USA) via EDC-NHS reaction. Qdot^®^ ITK™ carboxyl quantum dots are made from nanometer-scale crystals of a semiconductor material (CdSe), which are shelled with an additional semiconductor layer (ZnS). The size of QDs that were used in this study were within 15–20 nm.
(iii) gDNA hybridization: Five µL of the target gDNA in six size ranges (200–500 bp, 500–1000 bp, 1000–1500 bp, 1500–2000 bp, 2000–2500 bp, and 2500–3000 bp) with a negative control (water) and a positive control (unfragmented gDNA of *P. putida*, ~4 Mbp) were mixed with the probe and signaling complexes. Subsequently, a slow vertical rotation in a hybridization oven (UVP HB-500 Minidizer Hybridization, Fisher Scientific) was used to enable DNA hybridization overnight at 37 °C.
(iv) Fluorescence measurement: Following DNA hybridization, the hybridized complexes were washed three times using a phosphate buffer (pH = 7.4) to remove the untethered complexes. The endpoint fluorescence of QD_565_ and QD_655_ were measured using a SpectraMax M2 spectrofluorometer (Molecular Devices, Sunnyvale, CA, USA) at 570 and 660 nm, respectively, under excitation at 360 nm. All experiments were performed in triplicate, unless otherwise stated. The output of quantification was expressed by the ratio of the fluorescence intensity between QD_655_ and QD_565_ (QD_655_/QD_565_), as the signal (QD_655_) was normalized by the internal standard (QD_565_) to comprehend the different numbers of nanoparticles in each reaction.

### 2.4. Different Hybridization Duration Using Fragmented gDNA at Optimum Size Range

Different hybridization durations (0, 1, 2, 4, 8, and 12 h) were investigated using the optimum fragmented gDNA size range. The fragmented gDNA size range used was 1000–1500 bp. A total of 18 samples (triplicate for 6 time points) were prepared and used in the experiment. Three triplicate samples were taken out of the hybridization oven, washed with phosphate buffer three times, and transferred to a 96-well plate (Nunc, Roskilde, Denmark) at each time point. The fluorescence of the samples was measured using a SpectraMax M2 spectrofluorometer as described above.

### 2.5. Comparison of Fragmented (at Optimum Size Range) and Unfragmented gDNA via Quantification Capability of NanoGene Assay

Using a gDNA size range of 1000–1500 bp and 4 h of hybridization time, the NanoGene assay was performed with different amounts of target gDNA (0, 1, 4, 10, 40, 100, 200, and 400 ng). To compare, similar amounts of unfragmented gDNA were also used with a NanoGene assay.

## 3. Results and Discussion

### 3.1. Size Ranges of Fragmented gDNA

**Figure 2:**
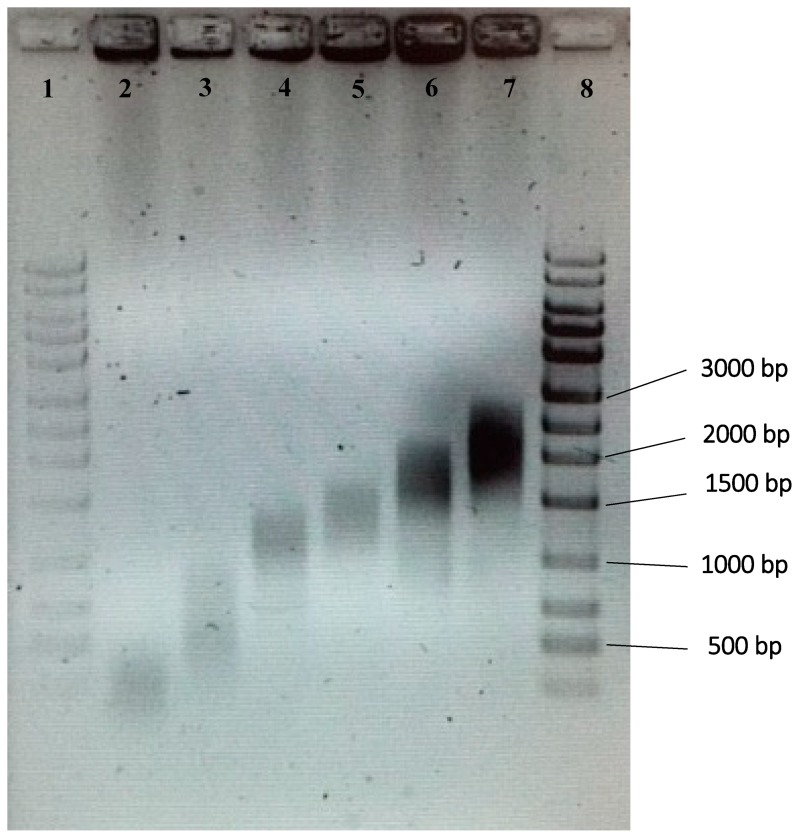
Gel photo of gDNA of various size ranges. Six different size ranges were used in the study. Lane 1, 100–bp ladder; lane 2, 200–500 bp; lane 3, 500–1000 bp; lane 4, 1000–1500 bp; lane 5, 1500–2000 bp; lane 6, 2000–2500 bp; lane 7, 2500–3000 bp; lane 8, 100–bp ladder.

Figure 2 shows the size ranges of gDNA fragments that were prepared via sonication followed by gel electrophoresis and extraction. Size ranges of 200–500 bp, 500–1000 bp, 1000–1500 bp, 1500–2000 bp, 2000–2500 bp, and 2500–3000 bp were collected and used in subsequent experiments to identify the optimum size range.

### 3.2. Quantification Capability of NanoGene Assay Using Fragmented gDNA of Different Size Ranges

**Figure 3:**
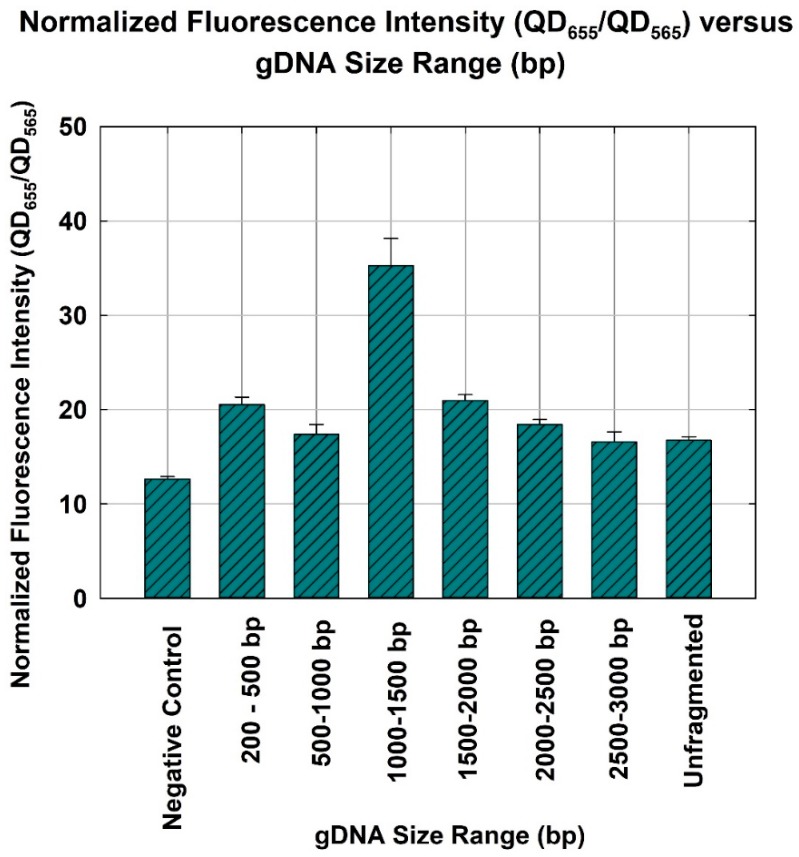
Gene quantification for *P. putida* detection, depicted by the normalized fluorescence intensity (QD_656_/QD_565_) of the NanoGene assay using gDNA of different size ranges. The bars and errors represent the mean and standard deviation of triplicate samples.

The negative control yielded a normalized fluorescence of 12.6 ± 0.263. As shown in Figure 3, the gDNA size range of 1000–1500 bp resulted in the highest normalized fluorescence (35 ± 2.895) and, therefore, corresponded to the highest quantification capability. In other words, the gDNA size range of 1000–1500 bp also yielded the highest hybridization efficiency and, therefore, was the optimum size range (*p*-value = 9.5 × 10^−12^, ANOVA). Pairwise *t* test was performed as a post-ANOVA, to show the statistical difference between the 1000–1500 bp gDNA and other sizes. As a result, all *P*-values of the *t* test (95% confidence level) between 1000–1500 bp, and all other fragmented DNAs (200–500 bp, 500–1000 bp, 1500–2000 bp, 2000–2500 bp, and 2500–3000 bp) were 0.005, 0.009, 0.004, 0.010, 0.008, and 0.004, respectively. This suggested all other fragmented DNA were statistically different from the 1000–1500 bp gDNA. Additionally, the normalized fluorescence of the unfragmented gDNA was similar to that of 2500–3000 bp gDNA. The *p*-value of *t* test comparing between unfragmented gDNA and 2500–3000 bp gDNA was 0.858 (95% confidence level). This suggested that gDNA with a size range equal to or larger than 2500 bp would behave like unfragmented gDNA during hybridization. As described in Figure 1, two DNA probes (i.e., DNA probe and signaling probe) that are used for the NanoGene assay were designed to target 306 bp of PAH-RHD_α_ gene of *P. putida* [22,23]. Given the target size of 306 bp, DNA templates of 200–500 bp or 500–1000 bp might not be sufficiently intact to ensure the binding of two DNA probes to the 306 bp target gene. On the other hand, DNA fragments that are 1500 bp or larger may still maintain the original supramolecular structure of gDNA and, thus, could lower the efficiency of DNA hybridization.

### 3.3. Different Incubation Duration Using Fragmented gDNA at Optimum Size Range

**Figure 4:**
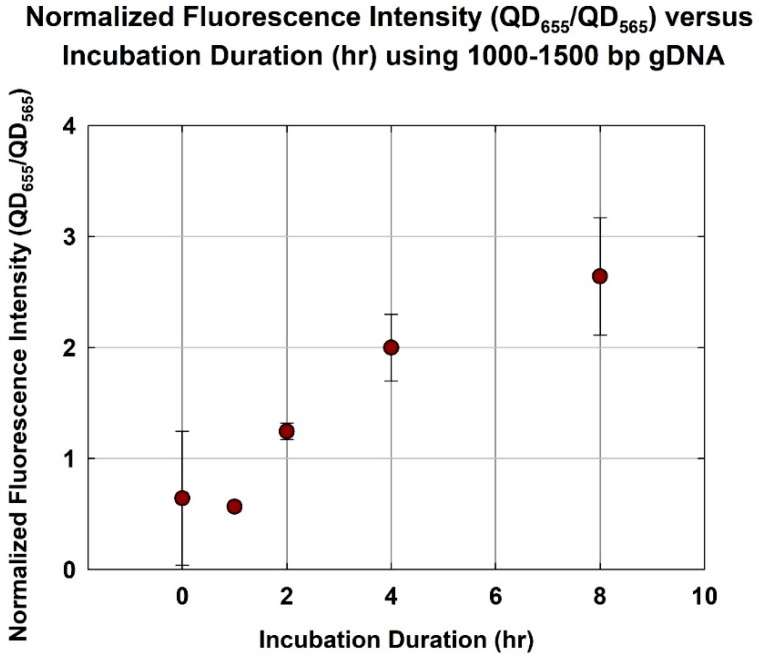
Normalized fluorescence intensity (QD_655_/QD_565_) versus incubation duration (h) using optimum size range gDNA (1000–1500 bp).

As shown in Figure 4, as expected, the normalized fluorescence increased with the incubation duration. The normalized fluorescence increased from 0.57 ± 0.038 to 2.0 ± 0.302 as the incubation duration increased from 1 h to 4 h. However, as the incubation duration increased from 4 to 8 h, the normalized fluorescence only increased marginally, from 2.0 ± 0.302 to 2.6 ± 0.528. In other words, hybridization appeared to have approached an asymptote at 4 h. This is consistent with earlier studies using unfragmented gDNA, which indicated that a minimum incubation duration of 8 h was needed [21,24]. Thus, a shorter gDNA at an optimum size range would hybridize significantly faster than unfragmented gDNA.

### 3.4. Comparison of Quantification Capability NanoGene Assay with Optimum gDNA Size Range and Unfragmented gDNA

**Figure 5:**
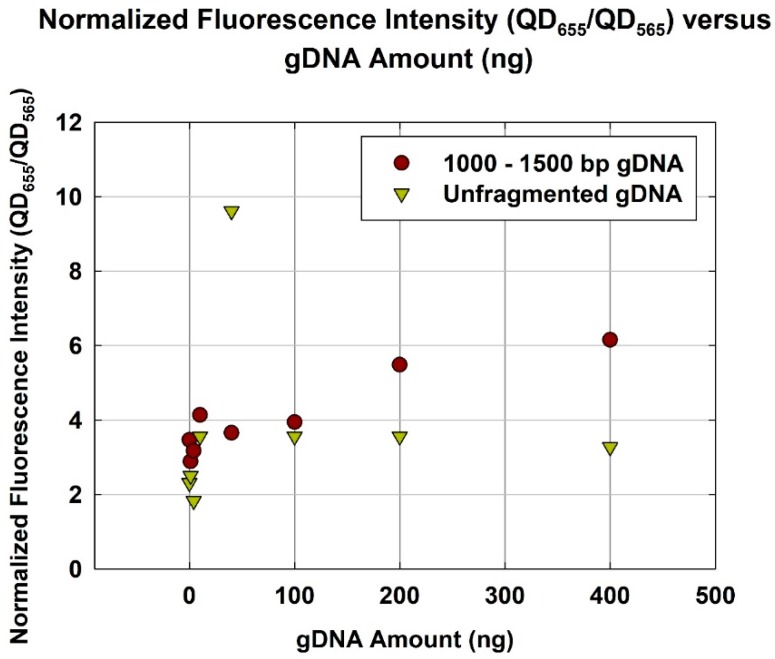
Normalized fluorescence intensity (QD_655_/QD_565_) versus gDNA amount (ng) for optimum size range (1000–1500 bp) and unfragmented gDNA.

Considering an incubation duration of 4 h, the gDNA with an optimum size range (1000–1500 bp) yielded a higher normalized fluorescence than unfragmented gDNA (Figure 5). Using 400 ng of gDNA, normalized fluorescence employing 1000–1500 bp gDNA was ~6.2, while that using unfragmented gDNA was ~3.3. The use of 1000–1500 bp gDNA almost double the normalized fluorescence. This is consistent, once again, with the hypothesis that there exists an optimum size range (1000–1500 bp) for the gDNA. Reaching the optimum size range, the quantification capability and, hence, hybridization efficiency, are greatly improved. This result indicates that the analytical sensitivity of gene quantification by the DNA hybridization-based assay can be improved by selecting the optimum template size.

The presented work describing the optimum size of a gDNA template and the accompanying incubation time is a part of the continuing efforts to complete the development of the NanoGene assay by optimizing conditions of DNA hybridization in particle suspension. DNA hybridization in the NanoGene assay was previously evaluated and optimized for buffer stringency, effects of additives, washing and passivation, and probe lengths and configuration [16,25,26]. Effects of reaction inhibitors were also investigated, and the following mitigation strategies were suggested for use with the environmental samples [18,27,28]. Organic matters and cations appeared to be the main inhibitors in the NanoGene assay, similar to the qPCR assay. This has allowed us to customize the pretreatment and lysis techniques for the NanoGene assay [21,23,29].

## 4. Conclusions

The authors have shown that the optimum size range of gDNA for the NanoGene assay was 1000–1500 bp. It yielded the highest normalized fluorescence at 35 ± 2.895, as compared to that of 200–500 bp at 20 ± 0.789, and 2500–3000 bp at 16 ± 1.053. Additionally, its quantification capability was almost twice that of unfragmented gDNA for a gDNA amount of 400 ng and incubation of 4 h. This study will allow for the improvement of the performance of NanoGene assay-based diagnostics in terms of kinetics and analytical sensitivity. Finally, an important implication of the result presented in this study is that it will become part of the custom optimized pretreatment process prior to DNA hybridization-based assays. Therefore, the improvement of DNA hybridization can be achieved through the use of size-specific fragmentation, rather than the existing procedure that involves whole gDNA and other supporting agents.
